# Optimizing supplemental light spectrum improves growth and yield of cut roses

**DOI:** 10.1038/s41598-023-48266-3

**Published:** 2023-12-04

**Authors:** Maryam Davarzani, Sasan Aliniaeifard, Mahboobeh Zare Mehrjerdi, Mahmood Reza Roozban, Seyyed Arash Saeedi, Nazim S. Gruda

**Affiliations:** 1 Photosynthesis Laboratory, Department of Horticulture, College of Agricultural Technology (Aburaihan), University of Tehran, Pakdasht, Tehran Iran; 2 Controlled Environment Agriculture Center, College of Agriculture and Natural Resources, University of Tehran, Tehran, Iran; 3 Department of Horticultural Science, INRES-Institute of Crop Science and Resource Conservation, University of Bonn, 53121 Bonn, Germany

**Keywords:** Photosystem II, Light responses

## Abstract

During the seasons with limited light intensity, reductions in growth, yield, and quality are challenging for commercial cut rose production in greenhouses. Using artificial supplemental light is recommended for maintaining commercial production in regions with limited light intensity. Nowadays, replacing traditional lighting sources with LEDs attracted lots of attention. Since red (R) and blue (B) light spectra present the important wavelengths for photosynthesis and growth, in the present study, different ratios of supplemental R and B lights, including 90% R: B 10% (R90B10), 80% R: 20% B (R80B20), 70% R: 30% B (R70B30) with an intensity of 150 µmol m^−2^ s^−1^ together with natural light and without supplemental light (control) were applied on two commercial rose cultivars. According to the obtained results, supplemental light improved growth, carbohydrate levels, photosynthesis capacity, and yield compared to the control. R90B10 in both cultivars reduced the time required for flowering compared to the control treatment. R90B10 and R80B20 obtained the highest number of harvested flower stems in both cultivars. Chlorophyll and carotenoid levels were the highest under control. They had a higher ratio of B light, while carbohydrate and anthocyanin contents increased by having a high ratio of R light in the supplemental light. Analysis of chlorophyll fluorescence was indicative of better photosynthetic performance under a high ratio of R light in the supplemental light. In conclusion, the R90B10 light regime is recommended as a suitable supplemental light recipe to improve growth and photosynthesis, accelerate flowering, and improve the yield and quality of cut roses.

## Introduction

Apart from morphological and signaling effects, photosynthesis of plants needs light as the source of energy^1^. Intensity, duration, and quality of light are determiant factors influencing photosynthesis and as the consequence growth and development of plants. Quality of light is attracted the attention of both scientists and industrialist in order to improve crop production with highest energy use efficiency in controlled environment agriculture (CEA)^2^. Nowadys by introduction of light-emitting diodes (LED) in horticulture, application of diverse light qualities on crops in CEA is possible^3^.

Light is an indispencible factors to drive efficient photosynthesis and to achieve optimum yield of crops. To boost photosynthesis and gurantee economical yield in areas where the natural environmental conditions do not provide enough sunlight, application of artificial light through supplemental lighting is a common practice^4,5^. Daily light integral (DLI) is an important factor in controlling the transition to flowering and flower production. During the winter season, due to the lack of natural light, the DLI would be dramatically reduced, which imposes serious challenges for cutting rose production. Increasing light intensity and DLI through supplemental light increases flower induction and growth^6^. Since the early thirties^7^ the possible use of supplementary light to increase the level of radiation was investigated to improve the yield of greenhouse roses^8^. The beneficial aspects of using supplemental light using high-pressure sodium (HPS) or fluorescent lamps have been reported a long time ago^9,10^.

Today, LEDs are used for lighting greenhouses because they are more efficient than traditional light sources such as HPS and metal halide lamps^11^. Light is the source of energy for driving photosynthesis and as a consequence, the growth of plants, for this reason, different characteristics of light such as spectrum, intensity, and photoperiod can be manipulated to optimize the lighting environment according to the purpose of producing products including increasing yield and maintaining quality. Photosynthesis is the pivotal plant process that is affected by red (R) and blue (B) wavelengths range of visible light^12^. Wavelengths in the R and B light ranges are mostly absorbed by chlorophyll pigments^13^. It has been reported that R light increases growth, biomass, and stem height through its effects on chloroplast development, photosynthetic functionality, electron transfer, etc.^14^. Inhibition of hypocotyl growth, control of flowering time, cotyledon development, light orientation, stomatal movements (stomatal opening) are among the effective roles of B light^15^. There are some challenges for using monochromatic lights for growing plants in controlled environment agriculture (CEA) systems. For example, the crops would be compacted with limited growth under monochromatic B light^16^, while, monochromatic R light induces morphological and photosynthetic abnormalities, termed R light syndrome^17^ and elongation^18^. Considering that the spectrum absorbed by leaves in the range of R and B light is about 90% of the overall absorbing spectrum of chlorophyll pigments, therefore these light spectra attracted lots of attention for controlled environment agriculture (CEA) systems^19^. It is worth noting that some beneficial responses have been also reported for the other light spectra. For instance, far red induces elongation and in some crops flower initiations^20^. However, still, R and B lights are the main spectra used as artificial light for crop production in CEA^17,21^.

Rose (*Rosa hybrida*) is considered as the most popular cut flower in the world^22^. In terms of production, it is at the top of cut flowers. This ecowithoutmic plant can be produced under controlled environments all year-round, but the yield and the quality of roses produced in seasons with limited light intensity (autumn and winter) are an economic challenge. Since there are universal agreements on the beneficial effects of R and B light spectral ranges on photosynthesis and crop growth, in this study these two wavelength ranges were used. However, the best combination of R and B light spectral ranges to be used as supplemental lighting of greenhouse crops has not been determined. Therefore, the present research was designed to find the most suitable supplementary light recipe for growth, yield, and quality of cut roses.

## Materials and methods

### Plant materials and growth condition

Two grafted commercial rose cultivars (ʻSamuraiʼ and ʻUtopiaʼ) propagated through the stenting method using *Rosa canina* as the scion part free from the virus were obtained from research greenhouse of Sharekord University, Shahrekord, Iran. Rooted samples were grown in pots with 12 cm height, 14 cm width of the opening, and 12 cm diameter of the bottom parts. Cocopeat and and perlite (3:1; v:v) were used to fill the pots in the research greenhouse of Horticulture Department, Agricultural Technology college, University of Tehran, Iran. Average day/night temperature of greenhouse was 25/18 ± 3 °C, and relative humidity of 50 ± 5% were kept inside the greenhouse. To feed the plants at vegetative stage, half strength of Hoagland's nutrient solution^23^ were used and at the start of flowering its full strength was used. This experiment started in a separate greenhouse for the proper application of treatments and better control of environmental conditions. The experiment continued for 6 months from September to March. The plant collection and use were in accordance with all the relevant guidelines.

### Applicatin of supplemental light

**Figure 1:**
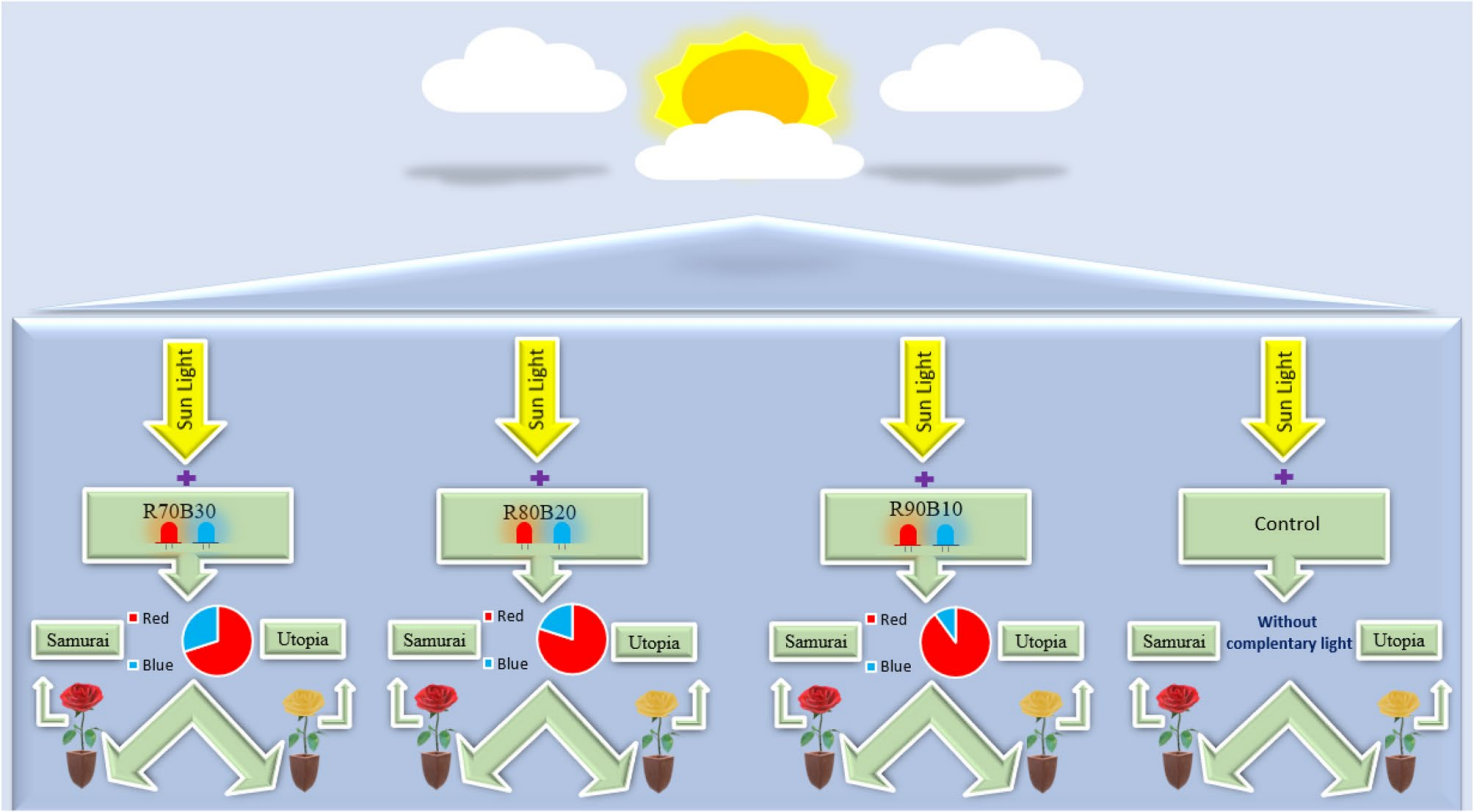
Schematic representation of the experimental setup for application of supplemental light with different quality of R and B lights for two commercial cut rose cultivars.

Supplemental lights that were added to the natural light included: 90% R: 10% B (R90B10), 80% R: 20% B (R80B20), 70% R: 30% B (R70B30) with an intensity of 150 µmol m^−2^ s^−1^ together with natural light, or without supplemental light (control) were investigated on two commercial rose cultivars namely ʻSamuraiʼ and ʻUtopiaʼ cultivars (Fig. 1). In this research, the main factor (supplementary light) and sub main factor (rose cultivars) were evaluated. Supplemental light treatments were applied using LED modules (Guangzhou Grow Light, 220–240 V, 18 W, 0.09 A). Supplemental lights were turned on for 12 h (7 AM to 7 PM). The light intensity was checked using a fluoropen device (FP 100‐MAX, Photon Systems Instruments, Drasov, Czech Republic), and the light spectrum was checked using a Sekonic Spectromaster (Sekonic C-7000, Japan). The experimental design was factorial experiment with 8 replications.

### Measurement of growth characteristics

**Figure 2:**
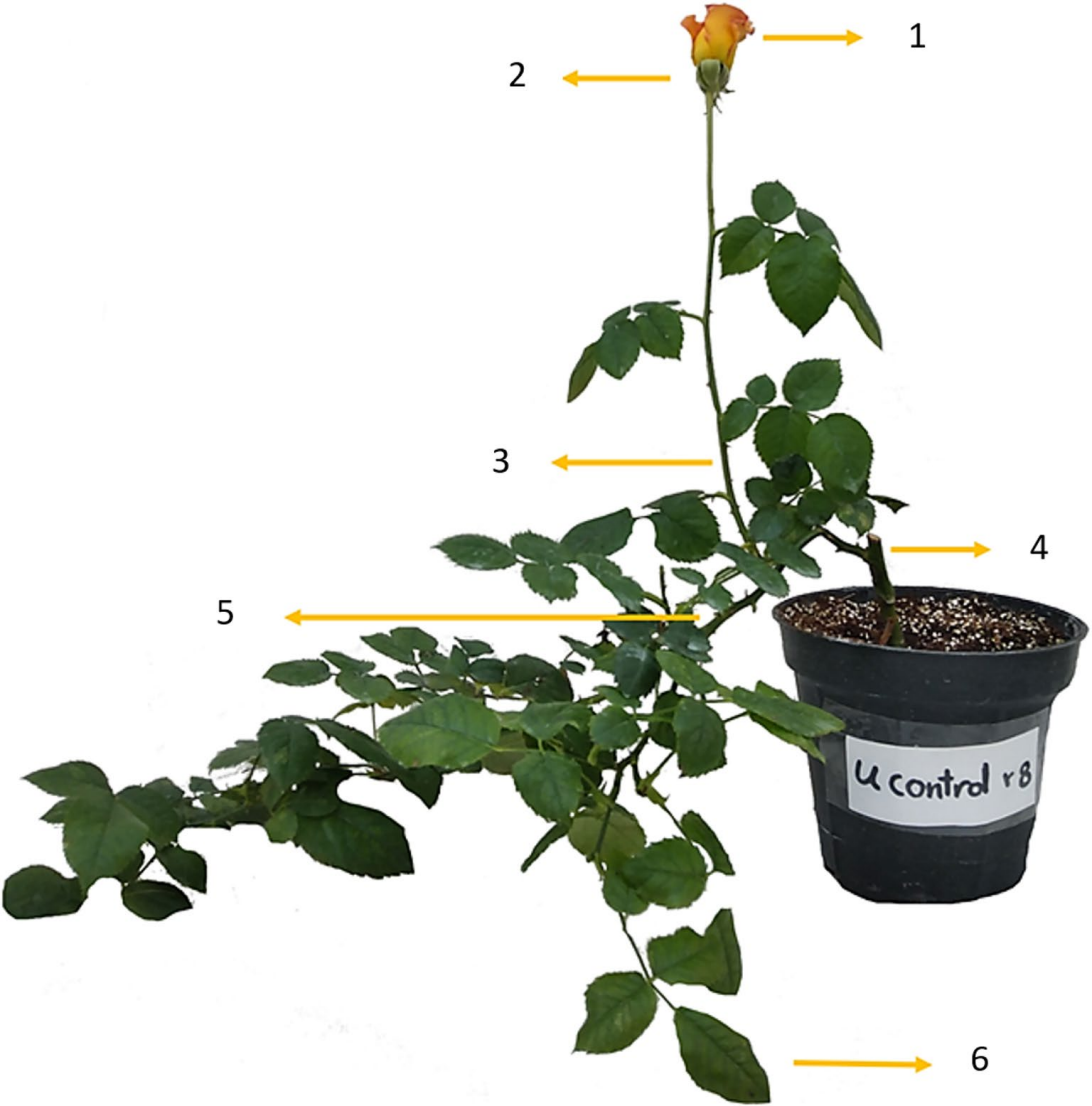
An illustration showing different parts of the rose crop that were used in the experiment. 1- Petal, 2- Receptacle, 3- Flowering stem, 4- Grafting site, 5- Bending shoot, and 6- Leaf.

In this study, a number of new shoots, shoot length, shoot thickness, leaf area, and specific leaf area were investigated in four replications. Following the growth of new shoots, all of them were bent at once, which is a common practice for training new roses. Thereafter, the number of new shoots was counted based on the shoots generated following the bending practice (Fig. 2).

At the end of the growing season, the length and thickness of bending shoots were evaluated. For this purpose, a ruler was used for the determination of shoot length with an accuracy of 1 mm, and a caliper was used for shoot thickness. To measure the fresh weight after separating each part from the plant, their weights were recorded. To measure the dry weight, after recording the fresh weight (the leaves were scanned after weighing), they were again weighed after 48 h in an oven at 72 °C. To measure the area of the leaves, a scanner device was used in such a way that all the leaves were separated from the stem and placed on the area of the scanner without overlapping. After scanning the leaves, Digimizer software (0.1.1.v4 Digimizer) was used to calculate the area.

After calculating the leaf area and leaf dry weight, specific leaf area (SLA) was obtained by dividing the leaf area by its dry weight. To measure the root traits, first, the roots were slowly removed from the bed and after removing the remaining cocopeat and perlite from them, the measurements were done. To obtain the root volume, a graduated cylinder containing a specific amount of water was used, and the difference in water volume in the presence of the root and before it was recorded as the root volume. Thereafter, the root dry weight was recorded following 48 h oven drying at 72 °C.

**Figure 3:**
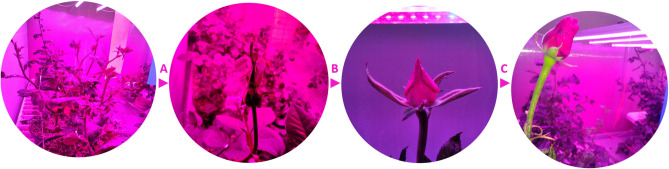
Following the appearance of the flowering stem, the flower bud developed (**A**), then it turned to red or pink color (**B**) and they were kept to grow to reach the appropriate stage of harvesting (**C**).

Reproductive characteristics including petal length, flower diameter, height of receptacle, receptacle diameter, stem length, and stem diameter, were investigated with eight replications. Also, number of cut flowers, emergence of flower buds, flower bud coloring, and flower harvest time were recorded using eight replications (Fig. 3). The average fresh and dry weight of each cut flower was determined following 48 h oven drying at 72 °C with four replications.

### Determination of pigments

Pigments including chlorophyll *a*, chlorophyll *b*, total chlorophyll, carotenoid, and anthocyanin, were measured with four replications. To measure chlorophyll and carotenoid contents, 0.1 g of fresh leaf tissue was weighed and ground in 2 mL of 96% ethanol. The resulting mixture was centrifuged at 13,000 rpm for 5 min. One mL of supernatant was used for reading in the spectrophotometer (Perkin Elmer Lambda 365 UV–Vis, Shelton, United States). At wavelengths of 666, 665, and 470 nm based on the method described by Lichtenthaler and Wellburn^24^. To measure the anthocyanin content, 20 mg of fresh plant tissue was rubbed with methanol acidified with 1% hydrochloric acid, and the resulting solution was placed in an incubator at 4 °C for 24 h. Following 24 h, the solution was centrifuged at 13,000 rpm for 15 min, and then the absorbance of the supernatant was read at 530 and 657 nm with a spectrophotometer^25^.

### Determination of carbohydrate contents

Carbohydrate content was measured with four replicates. To determine the soluble carbohydrates, therefore, 0.05 g of fresh leaf tissue was ground in a mortar, and 5 mL of 95% ethanol was added to it. Then the upper part of the solution was separated and the remaining sediments were washed again with five mL of 70% ethanol and the upper part was added to the previous solution. The extract was centrifuged for 15 min at 4500 rpm and after separating the upper phase, the resulting alcoholic extract was used for carbohydrate determination. To determine total soluble carbohydrates, 100 µL of the resulting extract was taken and 3 mL of anthrone (150 mg of pure anthrone + 10 mL of 72% sulfuric acid) was added to it. Then, it was placed in a hot water bath for 10 min. After cooling, the absorption at 625 nm wavelength was read with a spectrophotometer^26^. To prepare the standard curve, pure glucose with concentrations of 0, 100, 200, 300, 400, and 500 mg L^−1^ was used^27^.

For this purpose, the remaining sediments from the previous experiment were used to determine the stored carbohydrates. First, 5 mL of distilled water was added to the dried sediment. Then 6.5 mL of 52% perchloric acid was added to it and shaken for 15 min using a shaker. Then 20 mL of distilled water was added to it and centrifuged for 10 min at 6000 rpm. After removing the supernatant solution, the previous steps were repeated and the obtained solution was added to the previous solution. The solutions were filtered using filter paper and brought to a volume of 100 mL. Anthrone solution in 98% sulfuric acid was added and transferred to a bain-marie at 100 °C for 7.5 min. After the Ben-Marie time expired, the falcons were quickly transferred to the ice to cool down. Finally, reading was done using a spectrophotometer at a wavelength of 630 nm^27^.

### Chlorophyll fluorescence analysis

Analysis of chlorophyll fluorescence was carried out using Fluoropen and Fluorcam devices (Photon Systems Instruments, Brno, Czech Republic) based on the protocol for OJIP-transient and photosynthetic light curve. Measurements were carried out on fully-developed young leaves following 20 min of dark adaptation. The OJIP-transient test was performed to check the healthiness of the photosynthesis system under different light treatments. Calculations were done using PAR-Flourpen software. Based on the analysis of the measured fluorescence, the physiological state of the photosynthetic apparatus and the possible energy flow between the individual components of photosystem II were investigated (Table 1). A polyphasic chlorophyll fluorescence induction curve (O–J–I–P‐transient) was obtained in the leaves of each treatment. By employing the JIP test, the shape changes of the OJIP transient were quantitatively translated to a set of parameters, which relate to the in vivo adaptive behavior of the photosynthetic apparatus to the growth environment^17^. Measurements were conducted by using a PAR‐fluorPen FP 100‐MAX (Photon Systems Instruments) following dark adaptation (≥ 20 min). These were obtained at intervals of 50 µs, as well as of 3, 30, and 300 ms. The employed light intensity (3900 µmol m^−2^ s^−1^ photosynthetic photon flux density) was sufficient to generate maximal fluorescence for all treatments. Based on the OJIP protocol‐obtained data, the performance index for the photochemical activity (PI_ABS_) was calculated^28^. Light curves were also obtained by exposing the leaves to different photosynthetic photon flux densities [0 (darkness), 100, 200, 300, 500, and 1000 µmol m^−2^ s^−1^]. Due to the saturation of the light centers during the applied light intensities, the number of closed centers increases and there is a decreasing trend in the photosystem II efficiency. Eight leaves were assessed per treatment.

**Table 1:** Abbreviations and formulas of the parameters assessed based on the analysis of chlorophyll fluorescence.

PI_ABS_	Performance index for the photochemical activity	[(γRC/1 − γRC)(φ_P0_/1 − φ_P0_)(ψ_E0_/1 − ψ_E0_)]
Specific energy fluxes (per Q_A_ reducing PSII RC)		
ABS/RC	The specific energy fluxes per RC for energy absorption	M_0_ (1/V_J_)(1/)
TR_0_/RC	Trapped energy flux (leading to Q_A_ reduction) per RC	M_0_ (1/V_J_)
ET_0_/RC	Electron transport flux (further than Q_A_^−^) per RC	M_0_ (1/V_J_)(1 − V_J_)
DI_0_/RC	Dissipated energy flux	(ABS/RC) − (TR_0_/RC)
Basic parameters		
F_0_	Minimum fluorescence when all PSII reaction centers (RCs) are open (O-step of OJIP transient)	F_50µs_
F_J_	Fluorescence intensity at the J-step (2 ms) of OJIP	F_2ms_
F_I_	Fluorescence intensity at the I-step (30 ms) of OJIP	F_30ms_
Fluorescence parameters		
V_J_	Relative variable fluorescence at time 2 ms (J-step) after the start	(F_J_ − F_0_)/(F_m_ − F_0_)
F_m_	Maximum fluorescence, when all PSII RCs are closed (P-step of OJIP transient)	F_1s_ = F_p_

### Statistical analysis

There were eight plants in each treatment. However, 4–8 replications were used for each parameter, as indicated before. SAS 9.4 software was used to analyze the results of the experiments conducted in the present study. The mean comparison of traits was done using Duncan's test at the probability level of 5%.

## Results

### Vegetative and morphological analysis

**Figure 4:**
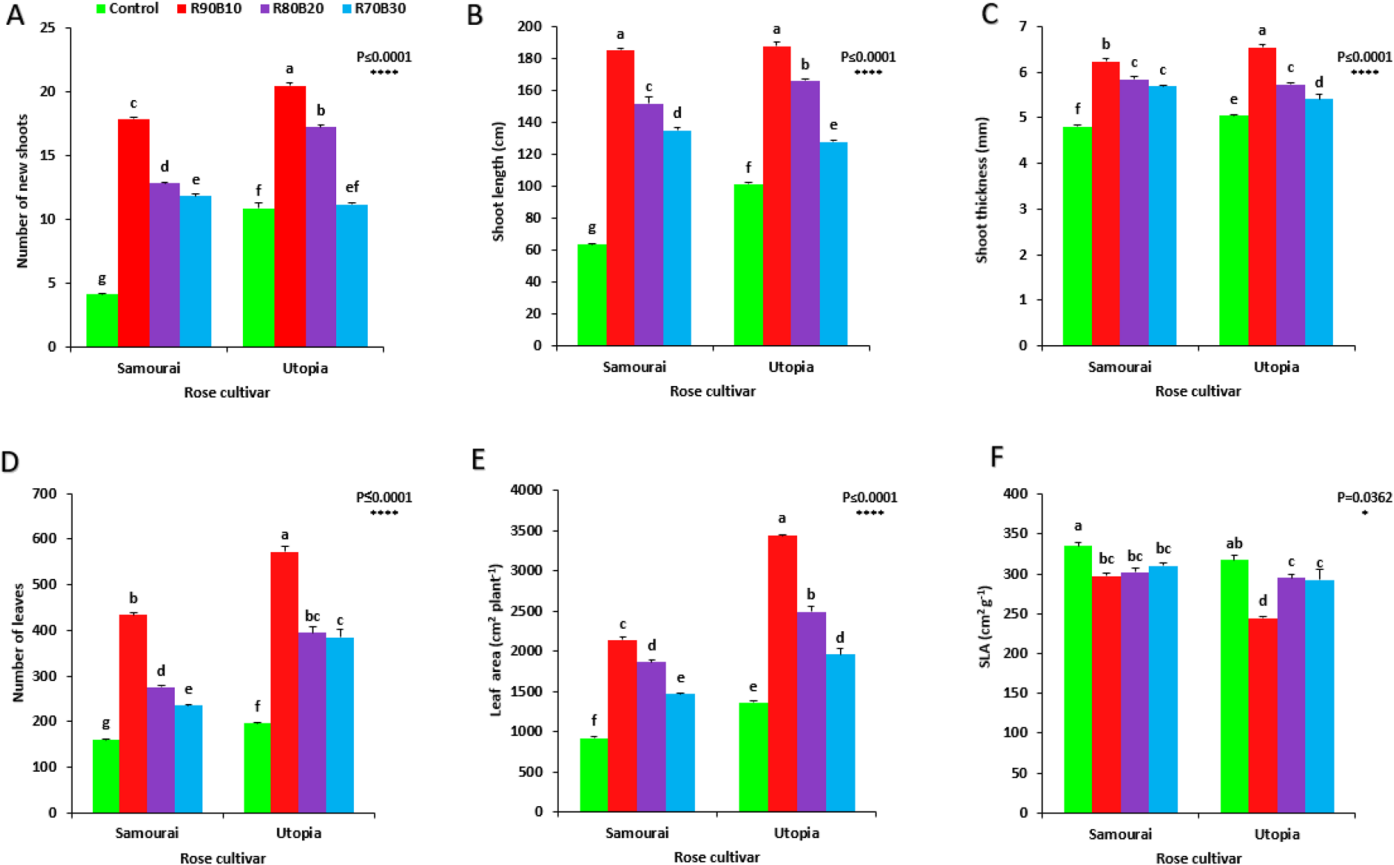
Effect of different ratios of R and B light spectra as supplemental light on the number of new shoots (**A**), shoot length (**B**), shoot thickness (**C**), number of leaves (**D**), leaf area (**E**) and specific leaf area (**F**) of two rose cultivars (ʻSamuraiʼ and ʻUtopiaʼ). Supplemental light recipes including 90% R: 10% B (R90B10), 80% R: 20% B (R80B20), 70% R: 30% B (R70B30), and treatment without supplement (Control) were used. Each column is representative of the mean value of four replicates plus SE. Significance at the 0.05 and 0.0001 probability levels are indicated by * and ****, respectively.

The results showed that the vegetative growth of two rose cultivars improved significantly with the use of supplemental light. The highest number of new shoots (20.52) was recorded under the R90B10 treatment on the ʻUtopiaʼ cultivar, while the lowest numbers of new shoots was recorded for the control treatment on the ʻSamuraiʼ cultivar (4.12 number). Among supplemental light recipes, increasing the ratio of B light decreased the number of newly emerged shoots in both rose cultivars (Fig. 4A). Tallest shoots were observed under R90B10 on ʻUtopiaʼ cultivar, while the shortest shoots were detected in control treatment of ʻSamuraiʼ cultivar. Among supplemental light recipes, increasing the ratio of B light decreased the shoot lenght in both rose cultivars (Fig. 4B). Thickest shoot diameter was observed under R90B10 treatment on ʻUtopiaʼ cultivar (6.55 mm), while, thinnest shoot diameter was detected in control treatment of ʻSamuraiʼ cultivar (4.81 mm). Among supplemental light recipes, increasing the ration of B light decreased the shoot thickness in both rose cultivars (Fig. 4C). Highest number of leaves was recorded under R90B10 treatment on the ʻUtopiaʼ cultivar (572.75 number), while, the lowest numbers of leaves was recorded for the control treatment on the ʻSamuraiʼ cultivar (160.75 number). Among supplemental light recipes, increasing the ratio of B light decreased the number of leaves in both rose cultivars (Fig. 4D). Widest leaf area was detected under R90B10 treatment on the ʻUtopiaʼ cultivar; while the lowest leaf area was observed in the control treatment of ʻSamuraiʼ cultivar. (Fig. 4E). Highest SLA was calculated for the plants under control treatment in both rose cultivars. ʻUtopiaʼ cultivar under R90B10 had the lowest SLA (Fig. 4F).

**Figure 5:**
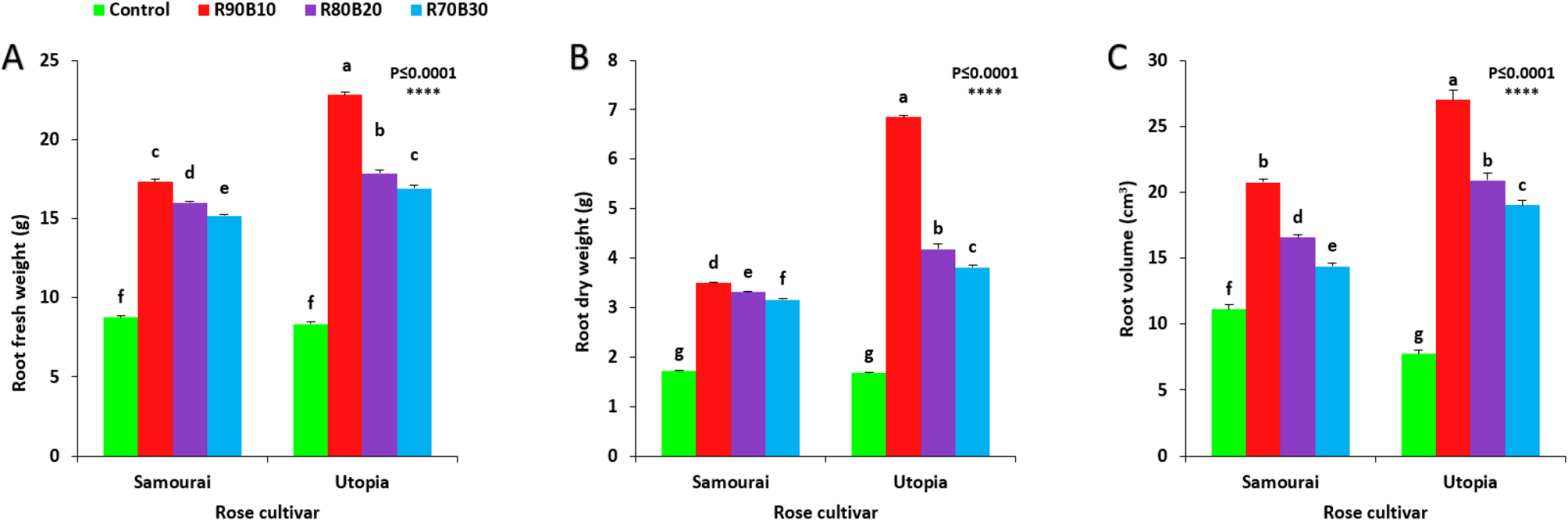
Effect of different ratios of R and B light spectrum as supplemental light on the root fresh weight (**A**), root dry weight (**B**), and root volume (**C**) of two rose cultivars (ʻSamuraiʼ and ʻUtopiaʼ). Supplemental light recipes including 90% R: 10% B (R90B10), 80% R: 20% B (R80B20), 70% R: 30% B (R70B30), and treatment without supplement (Control) were used. Each column is representative of the mean value of four replicates plus SE. Significance at the 0.0001 probability level is indicated by ****.

The highest root fresh weight (22.80 g) was observed under R90B10 treatment on the ʻUtopiaʼ cultivar, while the lowest root fresh weight was recorded for the control treatment on both rose cultivars (2.75 g). Among supplemental light recipes, increasing the ratio of B light decreased root fresh weight in both rose cultivars (Fig. 5A).

The highest root dry weight was observed under the R90B10 treatment on the ʻUtopiaʼ cultivar, while the lowest root dry weight was recorded for the control treatment in both rose cultivars. Among supplemental light recipes, increasing the ration of B light decreased root dry weight in both rose cultivars (Fig. 5B).

The highest root volume was observed under the R90B10 treatment on the ʻUtopiaʼ cultivar, while the lowest root volume was recorded for the control treatment in both rose cultivars. Among supplemental light recipes, increasing the ratio of B light decreased root volume in both rose cultivars (Fig. 5C).

### Biomass analysis

**Figure 6:**
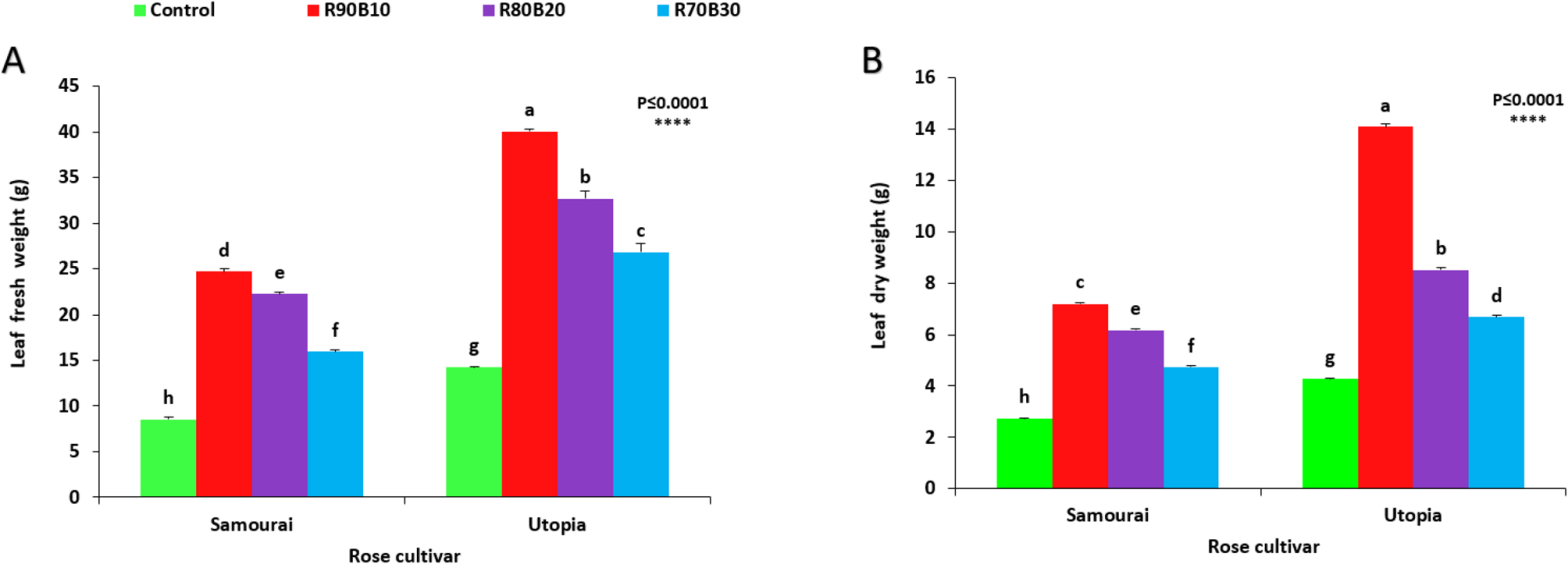
Effect of different ratios of R and B light spectra as supplemental light on the leaf fresh weight (**A**) and leaf dry weight (**B**) of two rose cultivars (ʻSamuraiʼ and ʻUtopiaʼ). Supplemental light recipes including 90% R: 10% B (R90B10), 80% R: 20% B (R80B20), 70% R: 30% B (R70B30), and treatment without supplement (Control) were used. Each column is representative of the mean value of four replicates plus SE. Significance at the 0.0001 probability level is indicated by ****.

The results showed that the biomass of two rose cultivars improved significantly with the use of supplemental light. The highest leaf fresh weight was achieved under the R90B10 treatment on ʻUtopiaʼ cultivar, while the lowest leaf fresh weight was recorded for the control treatment on the ʻSamuraiʼ cultivar (8.53 g). Among supplemental light recipes, increasing the ratio of B light decreased the leaf fresh weight in both rose cultivars (Fig. 6A). Highest leaf dry weight was achieved under R90B10 treatment on ʻUtopiaʼ cultivar (29.4 g), while the lowest leaf dry weight was recorded for the control treatment on the ʻSamuraiʼ cultivar (2.75 g). Among supplemental light recipes, increasing the ratio of B light decreased the leaf dry weight in both rose cultivars (Fig. 6B).

The mutual effect of supplementary light treatments and two rose cultivars was not significant, but a significant effect was observed among supplementary light treatments and also between two rose cultivars.

**Figure 7:**
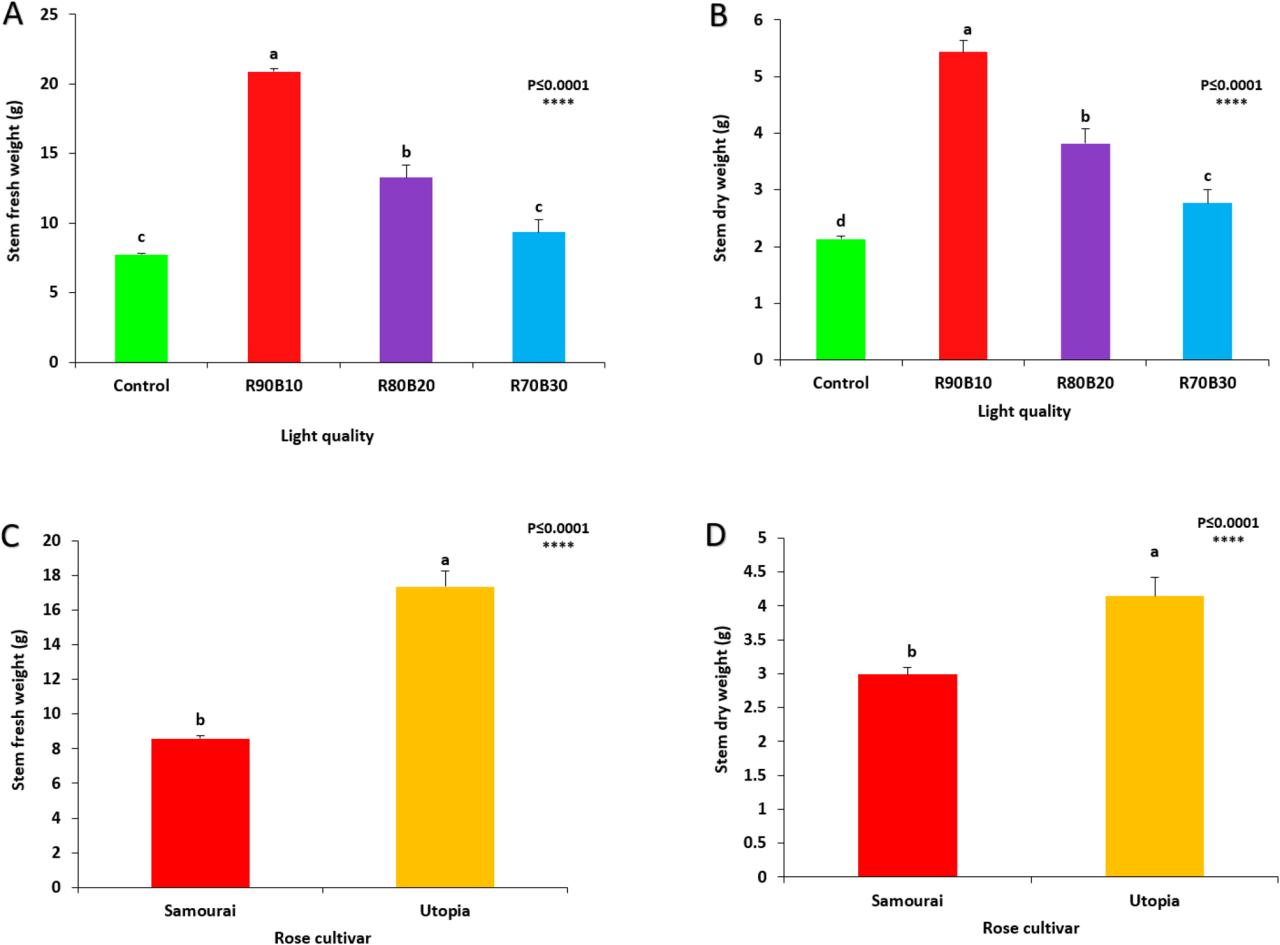
Effect of different ratios of R and B light spectra as supplemental light on the stem fresh weight of flower (**A**) and the stem dry weight of flower (**B**) also stem fresh weight of flower (**C**) and stem dry weight of flower (**D**) of two rose cultivars (ʻSamuraiʼ and ʻUtopiaʼ). Supplemental light recipes including 90% R: 10% B (R90B10), 80% R: 20% B (R80B20), 70% R: 30% B (R70B30), and treatment without supplement (Control) were used. Each column is representative of the mean value of four replicates plus SE. Significance at the 0.0001 probability level is indicated by ****.

The highest stem fresh weight was achieved under R90B10 treatment. In contrast, lowest stem fresh weight was recorded for control and R70B30 treatments (Fig. 7A). Among cultivars, ʻUtopiaʼ cultivar had more stem fresh weight than ʻSamuraiʼ (Fig. 7B).

The highest stem dry weight was calculated under R90B10 treatment. In contrast, lowest stem dry weight was recorded for the control treatment (Fig. 7C). Among cultivars, the ʻUtopiaʼ cultivar had more stem dry weight than ʻSamuraiʼ (Fig. 7D).

**Figure 8:**
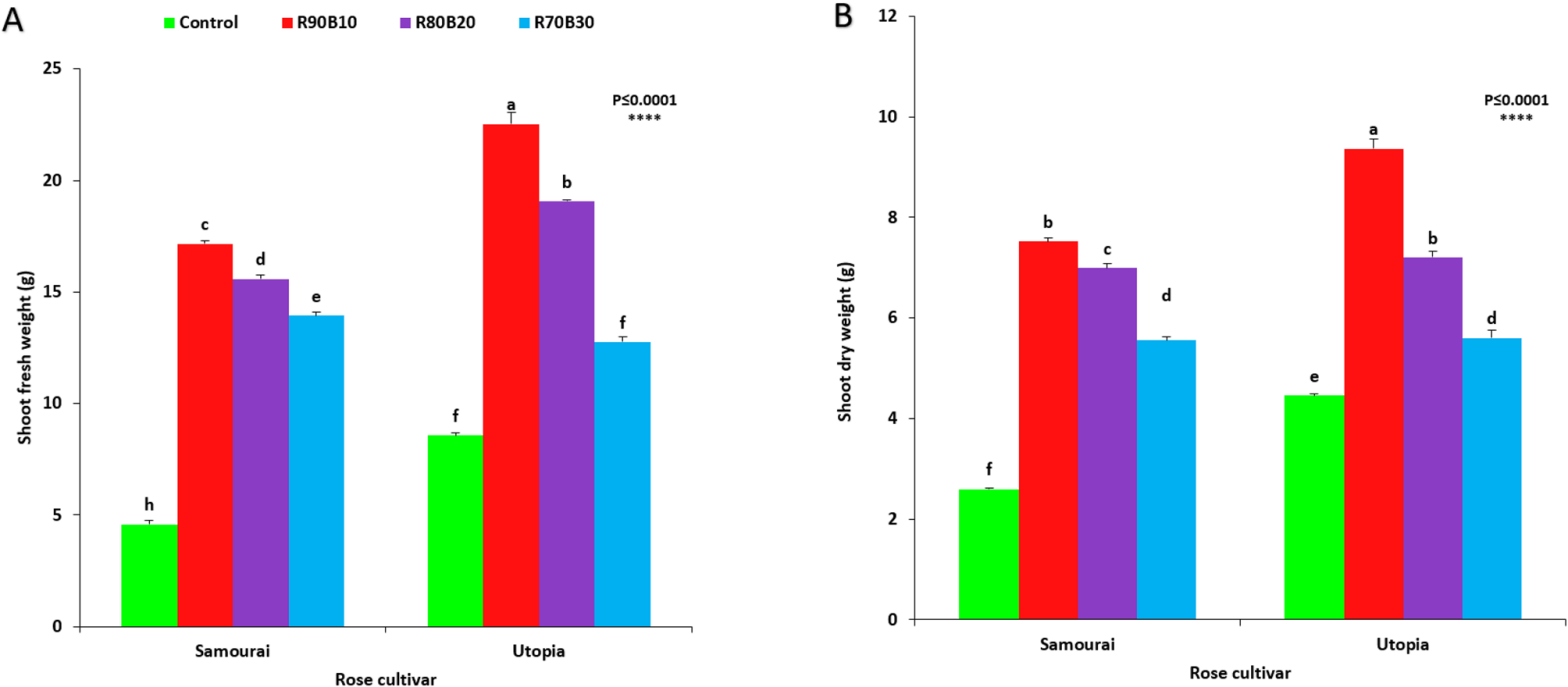
Effect of different ratios of R and B light spectra as supplemental light on the shoot fresh weight (**A**) and shoot dry weight (**B**) of two rose cultivars (ʻSamuraiʼ and ʻUtopiaʼ). Supplemental light recipes including 90% R: 10% B (R90B10), 80% R: 20% B (R80B20), 70% R: 30% B (R70B30), and treatment without supplement (Control) were used. Each column is representative of the mean value of four replicates plus SE. Significance at the 0.0001 probability level is indicated by ****.

The highest shoot fresh weight (22.52 g) was observed under R90B10 treatment on the ʻUtopiaʼ cultivar, while the lowest shoot fresh weight was recorded for the control treatment on the ʻSamuraiʼ cultivar (2.75 g). Among supplemental light recipes, increasing the ratio of B light decreased shoot fresh weight in both rose cultivars (Fig. 8A).

The highest shoot dry weight (9.36 g) was calculated under R90B10 treatment on the ʻUtopiaʼ cultivar, while the lowest shoot dry weight was recorded for the control treatment on the ʻSamuraiʼ cultivar (2.6 g). Among supplemental light recipes, increasing the ratio of B light decreased shoot dry weight in both rose cultivars (Fig. 8B).

The mutual effect of supplemental light treatments and two rose cultivars on flower dry and fresh weight was not significant, but a significant effect was observed among supplemental light treatments and also between two different cultivars.

**Figure 9:**
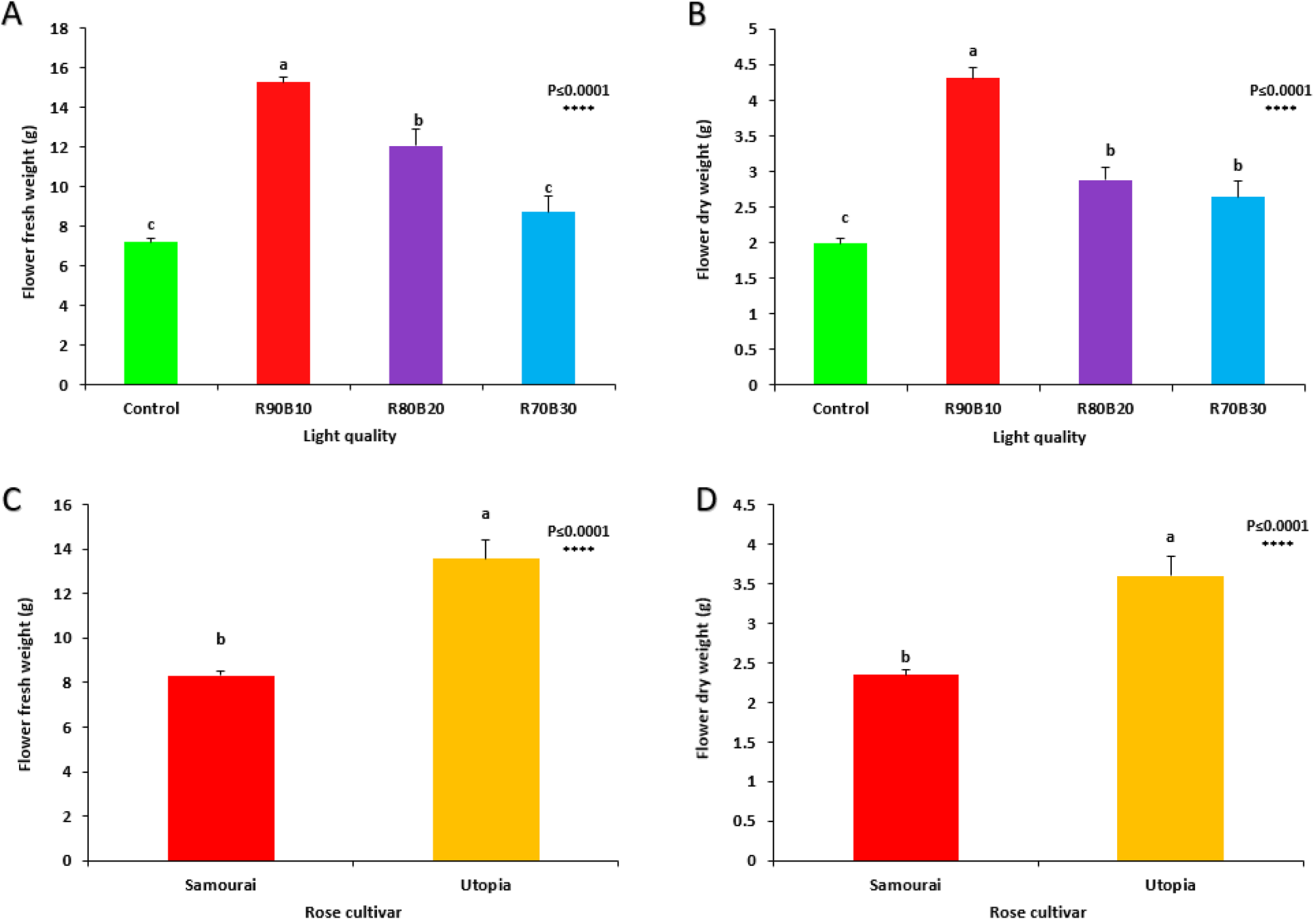
Effect of different ratios of R and B light spectra as supplemental light on the flower fresh weight (**A**) and the flower dry weight (**B**) and the effects of the cultivar (ʻSamuraiʼ and ʻUtopiaʼ) on the flower fresh weight (**C**) and flower dry weight (**D**). Supplemental light recipes including 90% R: 10% B (R90B10), 80% R: 20% B (R80B20), 70% R: 30% B (R70B30), and treatment without supplement (Control) were used. Each column is representative of the mean value of four replicates plus SE. Significance at the 0.0001 probability level is indicated by ****.

The highest flower fresh weight was achieved under the R90B10 treatment (15.29 g). In comparison, lowest flower fresh weight was recorded for control (7.21 g) and R70B30 (8.73 g) treatments (Fig. 9A). Among cultivars, ʻUtopiaʼ cultivar had more flower fresh weight than ʻSamuraiʼ (Fig. 9B).

The highest flower dry weight was calculated under R90B10 treatment (4.31 g). In comparison, lowest flower dry weight was recorded for the control (1.99 g) treatment (Fig. 9C). Among cultivars, ʻUtopiaʼ cultivar had more flower dry weight than ʻSamuraiʼ (Fig. 9D).

### Biomass partitioning

**Figure 10:**
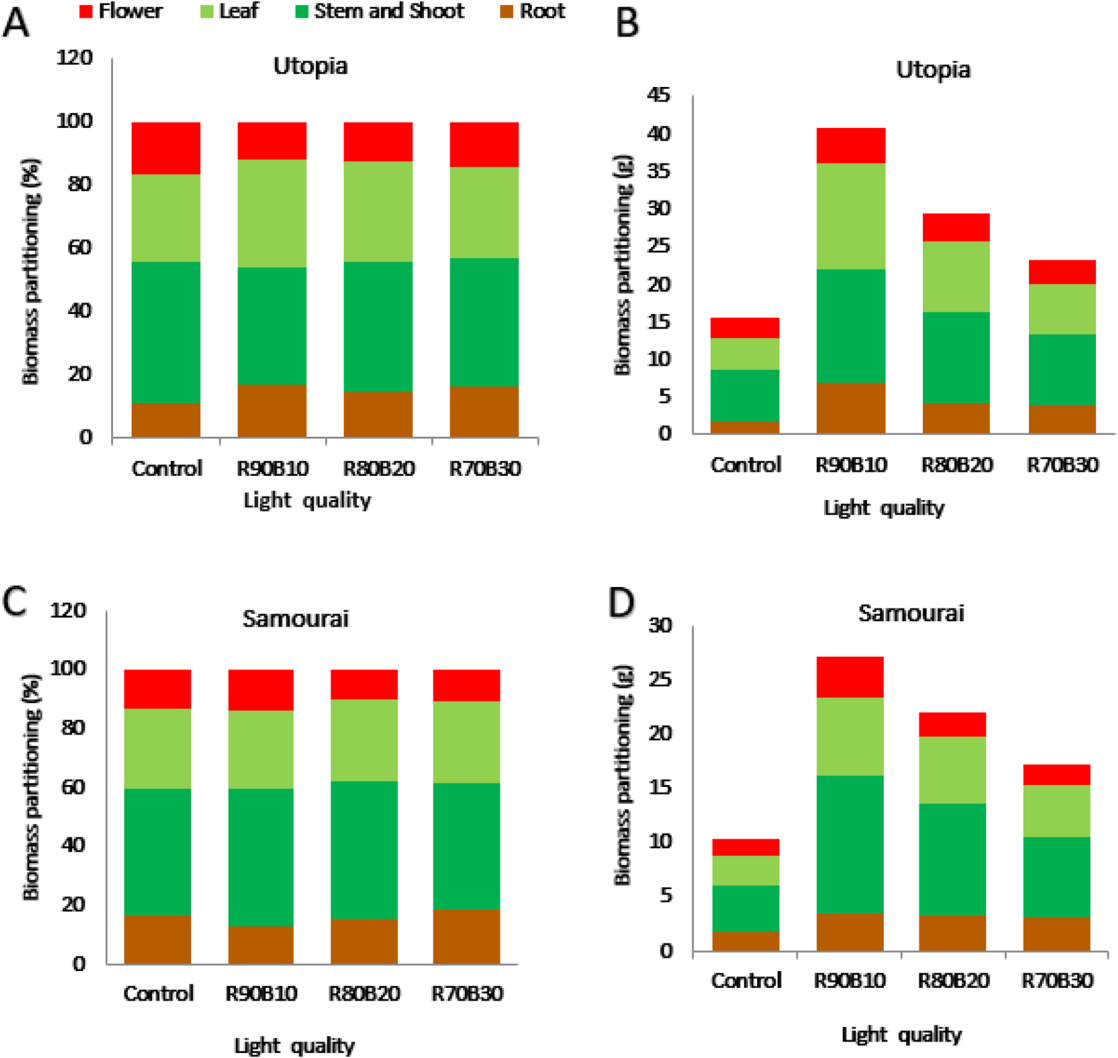
Effect of different ratios of R and B light spectra as supplemental light on biomass partitioning into the root, shoot, leaf, and flower based on percentage in total biomass (**A** and **C**) or weight of each part (**B** and **D**) of two rose cultivars (ʻSamuraiʼ and ʻUtopiaʼ). Supplemental light recipes including 90% R: 10% B (R90B10), 80% R: 20% B (R80B20), 70% R: 30% B (R70B30), and treatment without supplement (Control) were used.

The results showed that the biomass partitioning into the different organs of two rose cultivars was changed as a consequence of supplemental light application. Biomass partitioning of each cultivar into different parts including root, shoots, leaf, and flower was determined based on dry weight and percentage of each part in relation to the total weight of the plant. The stem and shoot are considered together as the shoot. In both cultivars, plants allocated most of their biomass into the leaf and shoot organs. The ʻUtopiaʼ cultivar had lower biomass for all the plant's organs under control conditions compared to the plants exposed to the supplemental light. This cultivar decreased the biomass partitioning into the root and increased biomass partitioning into the flowers. This can be an adaptive strategy to ensure survival under limited light conditions. The highest and lowest share of biomass partitioning into different organs under R90B10 treatment was calculated for shoot and flower, respectively. The highest and lowest share of biomass partitioning in control treatment was used for shoot and root, respectively. In the case of the ʻSamuraiʼ cultivar, it had lower biomass for all the plant's organs under control conditions compared to the plants exposed to the supplemental light. There was the almost the same share of biomass in the different organs when a comparison made between control and supplemental light treatments. By increasing the ratio of B light, there was a decrease in biomass allocation to the floral organs was detected (Fig. 10).

### Rose flower morphology

**Figure 11:**
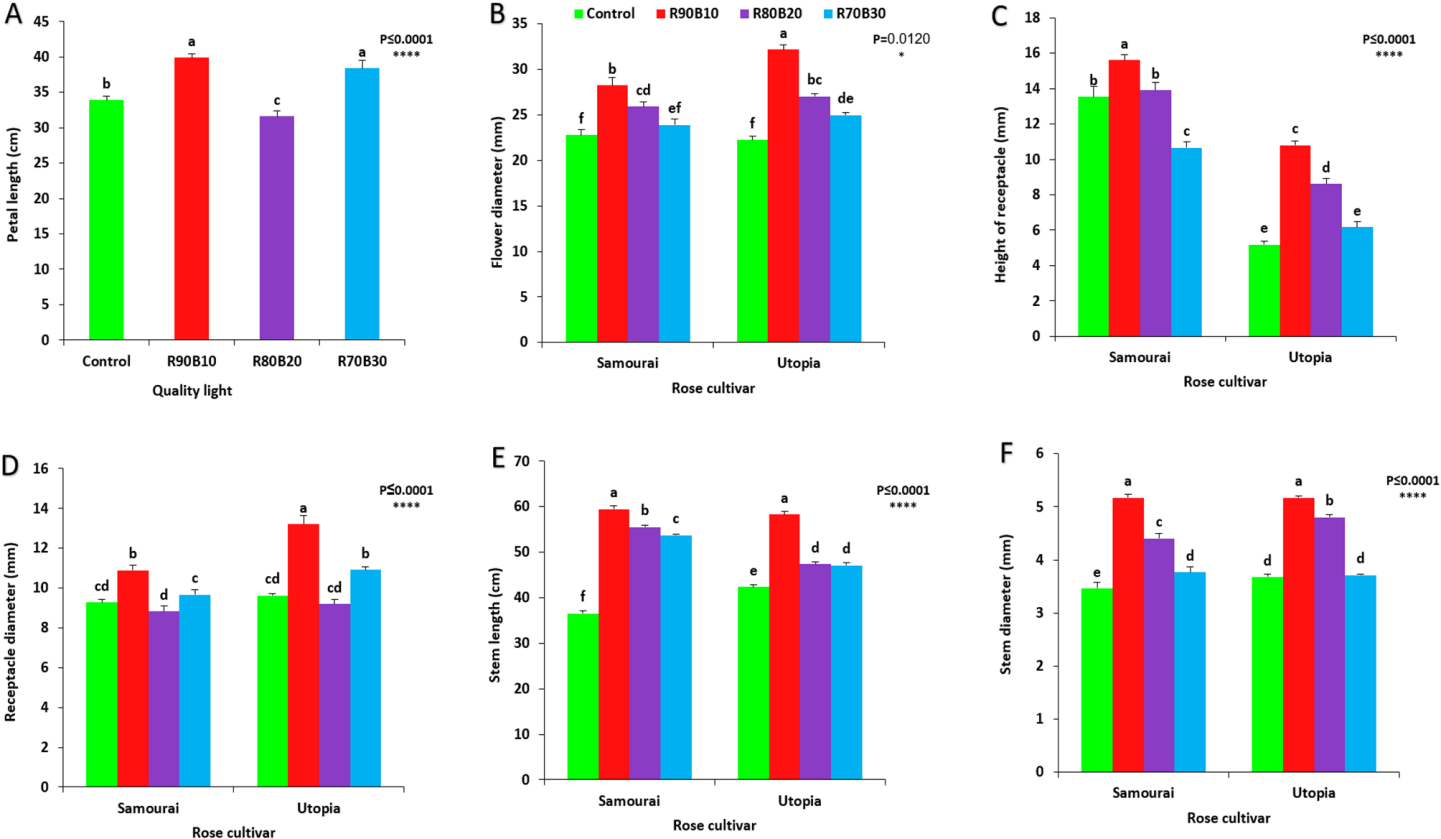
Effect of different ratios of R and B light spectra as supplemental light on the petal length (**A**), flower diameter (**B**), height of receptacle (**C**), receptacle diameter (**D**), stem length (**E**) and stem diameter (**F**) of two rose cultivars (ʻSamuraiʼ and ʻUtopiaʼ). Supplemental light recipes including 90% R: 10% B (R90B10), 80% R: 20% B (R80B20), 70% R: 30% B (R70B30), and treatment without supplement (Control) were used. Each column is representative of mean value of four replicates plus SE. Significance at the 0.05 and 0.0001 probability levels are indicated by * and ****, respectively.

The results showed that the rose floral morphology was improved significantly with the use of supplemental light. The tallest petal were observed under R90B10 (39.88 mm) and R70B30 (39.36 mm) treatments, while the shortest petal was detected under R80B10 (31 mm) treatment (Fig. 11A).

Thickest flower diameter was observed under R90B10 treatment on the ʻUtopiaʼ cultivar (32.16 mm), while, the thinnest flower diameter was detected in control treatment in both rose cultivars (Fig. 11B).

The longest receptacle was observed under R90B10 treatment on ʻSamuraiʼ cultivar. The lowest numbers of new shoots was recorded for the control treatment on the ʻUtopiaʼ cultivar (Fig. 11C). Thickest receptacle was observed under R90B10 treatment on the ʻUtopiaʼ cultivar (Fig. 11D).

The tallest stems were observed under R90B10 treatment on both rose cultivars, while the shortest stem was detected in control treatment on the ʻSamuraiʼ cultivar (Fig. 11E).

The thickest stem diameter was observed under R90B10 treatment on both rose cultivars, while, the thinnest stem diameter was detected in control treatment on the ʻSamuraiʼ cultivar (Fig. 11F).

### Flowering time, yield, and morphology

Flowering, yield, and morphology of the two studied rose cultivars were improved significantly by the use of supplemental light.

**Figure 12:**
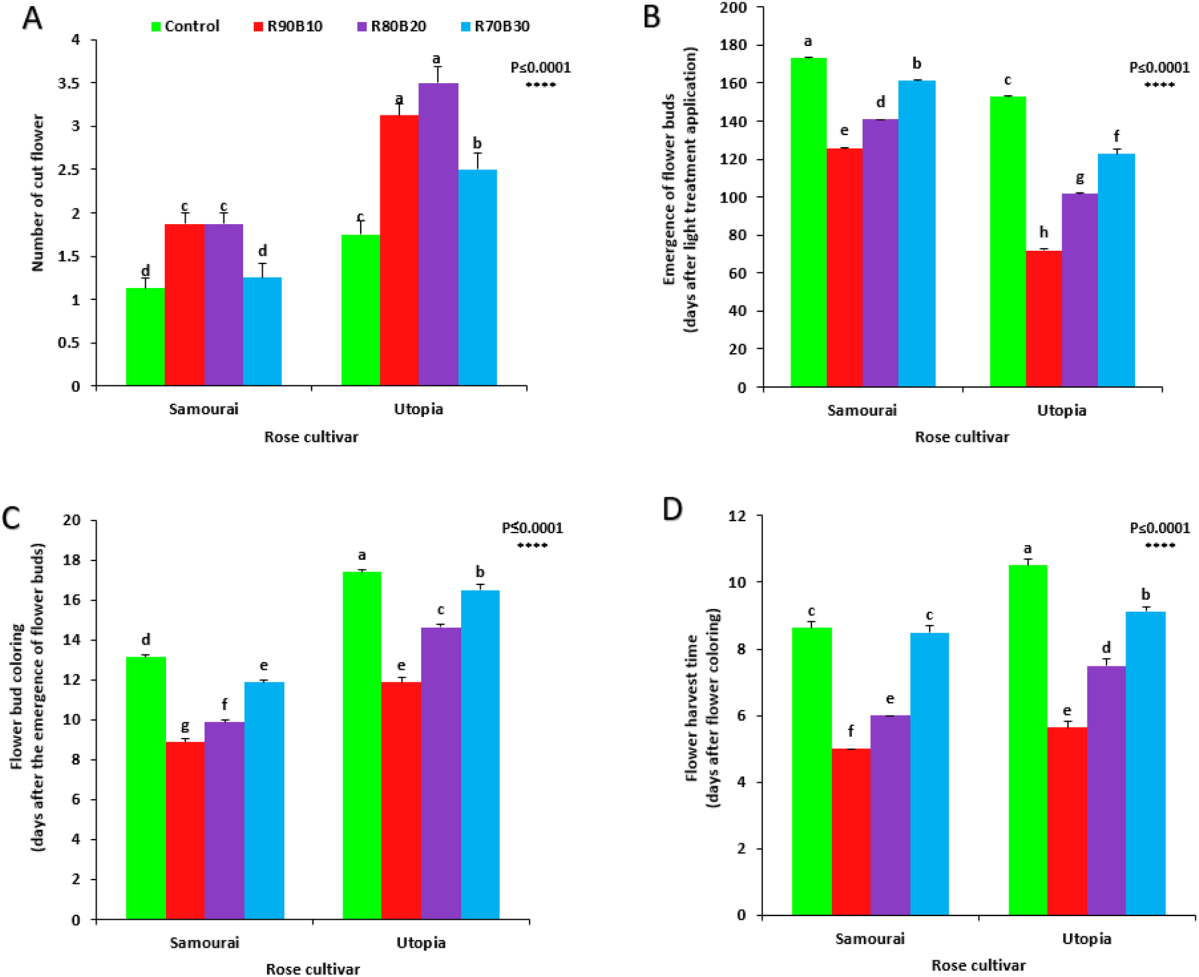
Effect of different ratios of R and B light spectra as supplemental light on the number of cut flower (**A**), emergence of flower bud (**B**), Flower bud coloring (**C**), and flower harvest time (**D**) of two rose cultivars (ʻSamuraiʼ and ʻUtopiaʼ). Supplemental light recipes including 90% R: 10% B (R90B10), 80% R: 20% B (R80B20), 70% R: 30% B (R70B30), and treatment without supplement (Control) were used. Each column is representative of the mean value of four replicates plus SE. Significance at the 0.0001 probability level is indicated by ****.

Highest number of cut flowers was recorded under R90B10 and R80B20 treatments on the ʻUtopiaʼ cultivar, while, the lowest number of cut flower was recorded for the control and R70B30 treatments on the ʻSamuraiʼ cultivar (Fig. 12A).

Under control conditions, both cultivars spent longer time for the emergence of their first bud than the time needed for first bud emergence under supplemental light treatments and by increasing the ratio of R to B light, the time required for the appearance of the first buds decreased. The fastest first bud emergence belonged to the ʻUtopiaʼ cultivar under R90B10, which takes in average of 62.2 days for its first bud appearence (Fig. 12B).

The longest duration for flower bud coloring was detected under control treatment on ʻUtopiaʼ cultivar, while shortest time for flower bud coloring was recorded for the R90B10 treatment on the ʻSamuraiʼ cultivar (Fig. 12C). Longest duration needed to harvest the flower was recorded under control treatment on ʻUtopiaʼ cultivar, while shortest duration needed to harvest the flower was recorded for the R90B10 treatment on the ʻSamuraiʼ cultivar (Fig. 12D).

**Figure 13:**
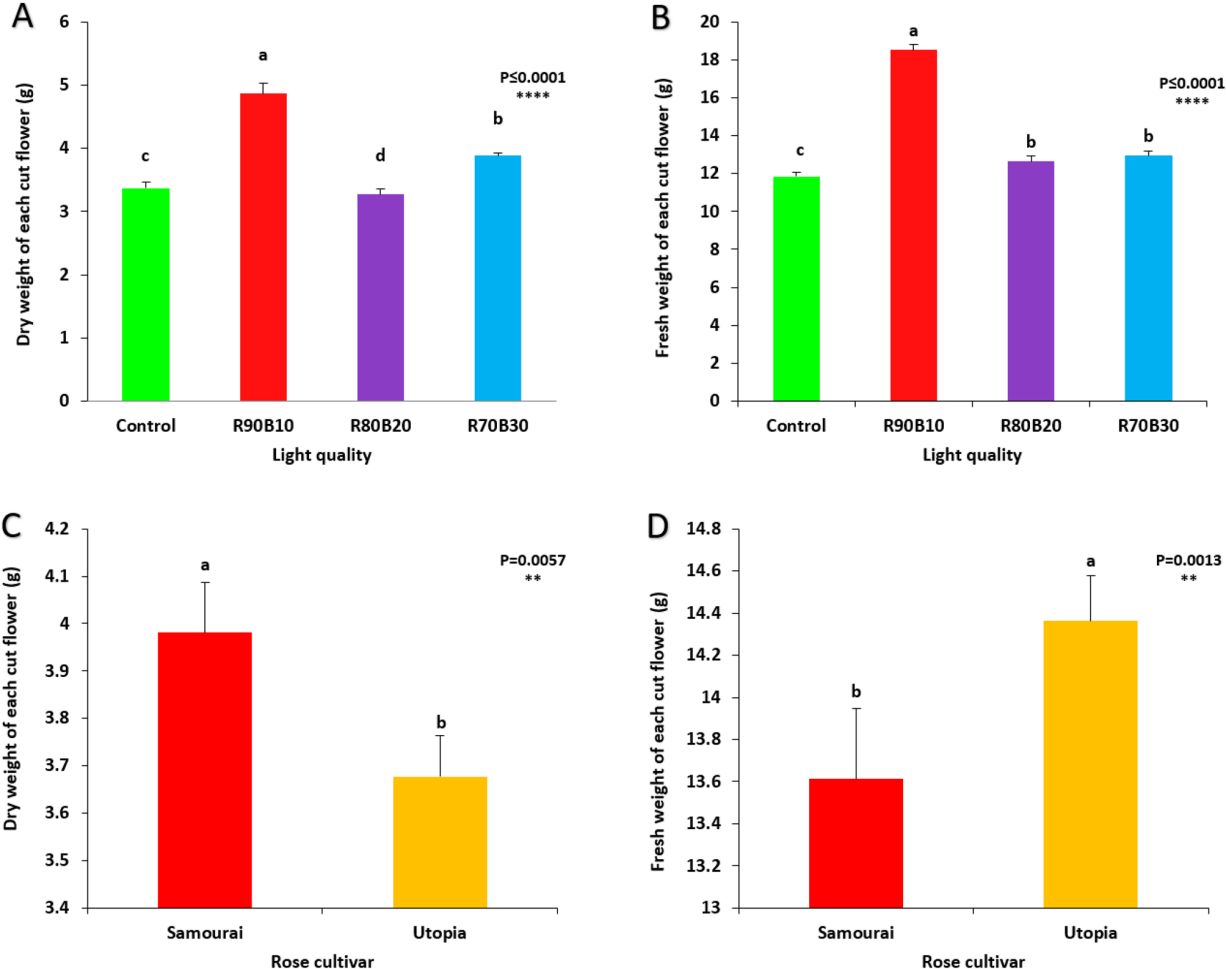
Effect of different ratios of R and B light spectra as supplemental light on the average dry weight (**A**) and fresh weight (**B**) of a rose and also the effect of two rose cultivars on the average dry weight (**C**) and fresh weight (**D**) of two rose cultivars (ʻSamuraiʼ and ʻUtopiaʼ). Supplemental light recipes including 90% R: 10% B (R90B10), 80% R: 20% B (R80B20), 70% R: 30% B (R70B30), and treatment without supplement (Control) were used. Each column is representative of the mean value of four replicates plus SE. Significance at the 0.01 and 0.0001 probability levels are indicated by ** and ****, respectively.

The mutual effect of supplementary light treatments and two rose cultivars on dry and fresh weight of each cut flower fresh weight than ʻSamuraiʼ (Fig. 13D). was not significant, but a significant effect was observed among supplementary light treatments and also between two different cultivars.

The highest dry weight of each cut flower was achieved under the R90B10 treatment. In contrast, lowest dry weight of each cut flower was recorded for R80B20 treatment (Fig. 13A). Among cultivars, ʻSamuraiʼ cultivar had more dry weight for each cut flower than ʻUtopiaʼ (Fig. 13B).

The highest flower fresh weight of each cut flower was calculated under the R90B10 treatment. In contrast, lowest flower fresh weight was recorded for the control treatment (Fig. 13C). Among cultivars, ʻUtopiaʼ cultivar had more flowers.

### Pigment and carbohydrate analysis

**Figure 14:**
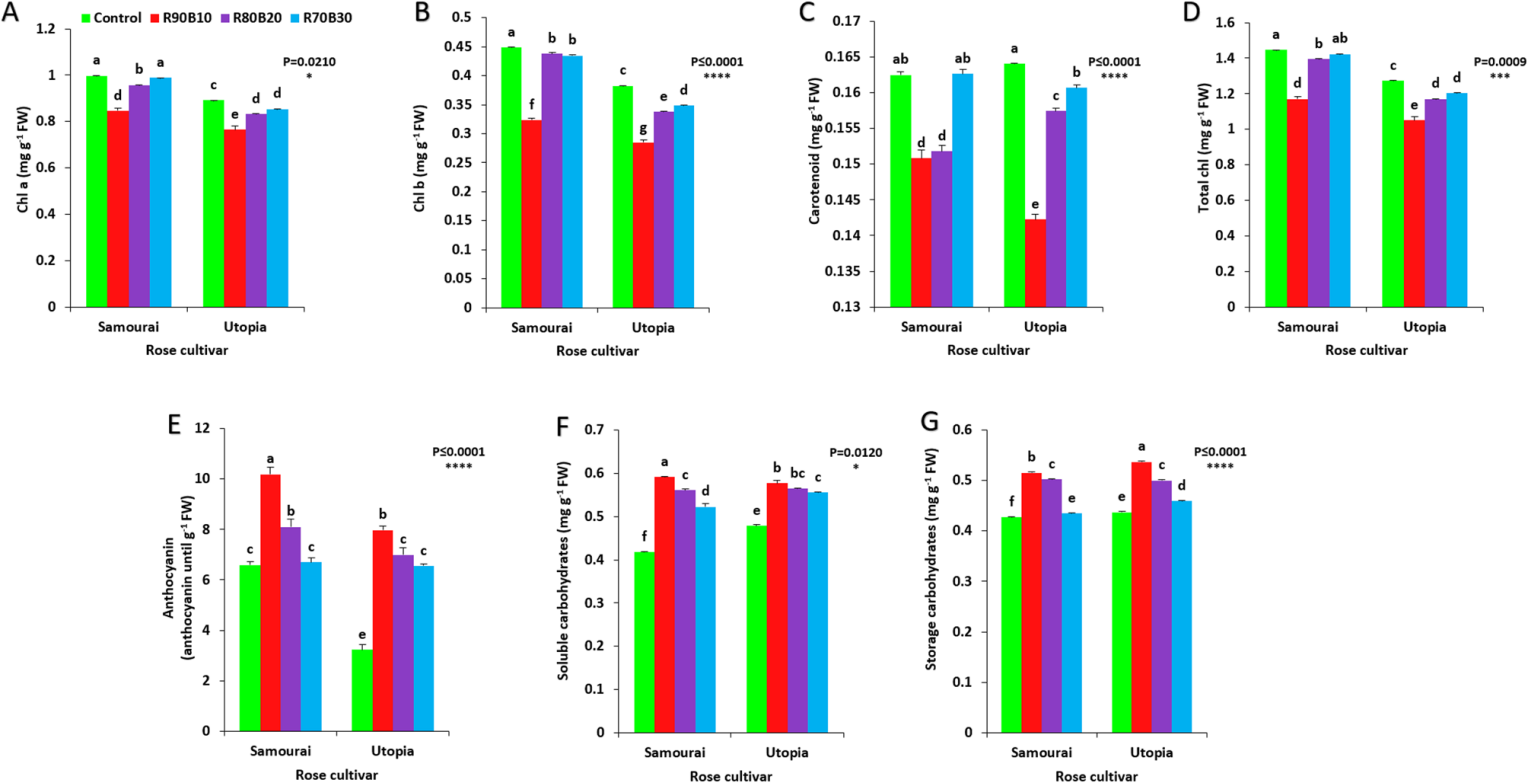
Effect of different ratios of R and B light spectra as supplemental light on chlorophyll a (**A**), chlorophyll b (**B**), carotenoids (**C**), total chlorophyll (**D**), anthocyanin (**E**), soluble carbohydrates (**F**) and storage carbohydrates (**G**) of two rose cultivars (ʻSamuraiʼ and ʻUtopiaʼ). Supplemental light recipes including 90% R: 10% B (R90B10), 80% R: 20% B (R80B20), 70% R: 30% B (R70B30), and treatment without supplement (Control) were used. Each column is representative of the mean value of four replicates plus SE. Significance at the 0.05, 0.001, and 0.0001 probability levels are indicated by *, ***, and ****, respectively.

The pigment and carbohydrate of the two studied rose cultivars were significantly affected by the use of supplementary light. The highest concentration of chlorophyll *a* was detected under control and R70B30 treatments on the ʻSamuraiʼ cultivar, while the lowest chlorophyll *a* concentration was recorded for the R90B10 on ʻUtopiaʼ cultivar (Fig. 14A).

The highest concentration of chlorophyll *b* was detected under control treatment on the ʻSamuraiʼ cultivar, while lowest chlorophyll *b* concentration was recorded for the R90B10 treatment on the ʻUtopiaʼ cultivar (Fig. 14B). Lowest carotenoids was achieved in the R90B10 treatment on ʻUtopiaʼ cultivar (Fig. 14C).

The highest total chlorophyll was detected under control treatment on the ʻSamuraiʼ cultivar, while the lowest total chlorophyll was recorded for the R90B10 treatment on the ʻUtopiaʼ cultivar (Fig. 14D).

The highest anthocyanin was detected under R90B10 treatment on the ʻSamuraiʼ cultivar, while the lowest anthocyanin was recorded for the control treatment on the ʻUtopiaʼ cultivar (Fig. 14E).

The highest soluble carbohydrates were measured under the R90B10 treatment on the ʻSamuraiʼ cultivar, while the lowest soluble carbohydrates were recorded for the control treatment on the ʻSamuraiʼ cultivar (Fig. 14F). Highest storage carbohydrates were observed under R90B10 treatment on ʻUtopiaʼ cultivar, while lowest storage carbohydrates was recorded for the control treatment on ʻSamuraiʼ cultivar (Fig. 14G).

### Chlorophyll fluorescence analysis

The results showed that chlorophyll fluorescence parameters were significantly affected by supplemental light treatments.

**Figure 15:**
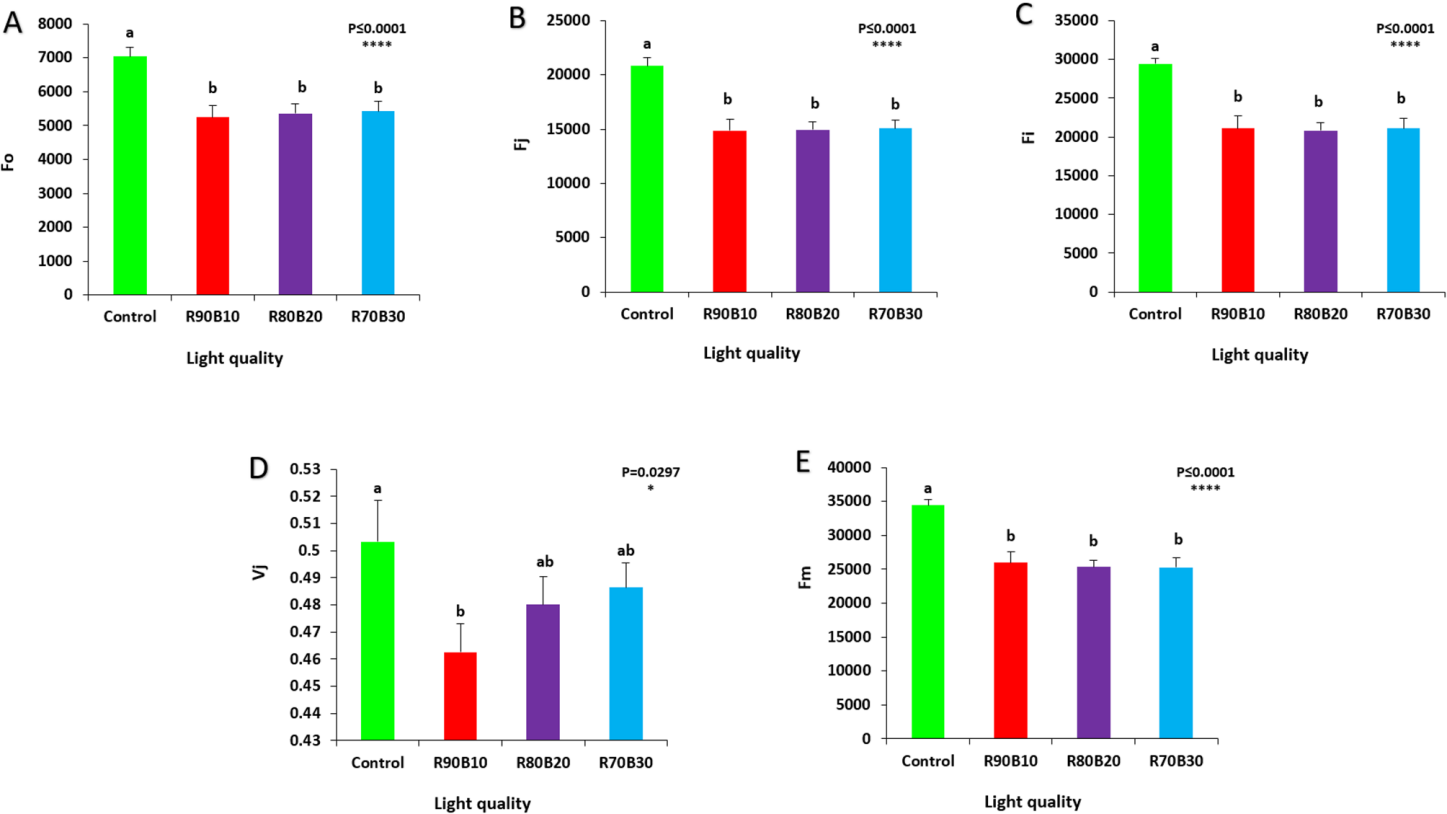
Effect of different ratios of R and B light spectrum as supplemental light on F_o_ (minimal fluorescence yield of the dark-adapted state) (**A**), F_j_ (Fluorescence intensity at the J-step (2 ms) of OJIP) (**B**), F_i_ (Fluorescence intensity at the I-step (30 ms) of OJIP) (**C**), V_j_ (relative variable fluorescence at J-step) (**D**) and F_m_ (maximal fluorescence of the dark-adapted state) (**E**) of two rose cultivars (ʻSamuraiʼ and ʻUtopiaʼ). Supplemental light recipes including 90% R: 10% B (R90B10), 80% R: 20% B (R80B20), 70% R: 30% B (R70B30), and treatment without supplement (Control) were used. Each column is representative of the mean value of four replicates plus SE. Significance at the 0.05 and 0.0001 probability levels are indicated by * and ****, respectively.

Fluorescence emission parameters detected from the chlorophyll fluorescence analysis including F_0_, F_i_, F_j_, and F_m_ were the highest under control conditions. Supplemental light applications caused a significant decrease in those parameters compared to the corresponding values in Control (Fig. 15A,B,C,E). The highest V_j_ was detected under the Control treatment, while the lowest V_j_ was recorded for the R90B10 treatment (Fig. 15D).

The highest PI_ABS_ was recorded under the R90B10 treatment, while the lowest PI_ABS_ was recorded for the control treatment (Fig. S1A). ABS/RC, TR_0_/RC, ET_0_/RC, and DI_0_/RC were the highest under control conditions and supplemental light applications caused a significant decrease in those parameters compared to the control (Fig. S1B,C,D,E).

## Discussion

### Supplemental light with a high ratio of R light improved the growth traits of roses

An essential factor that need to be optimized for the growth and development of greenhouse crops is the lighting environment^14^. The use of supplemental light to increase the productivity of roses has been widely investigated and is even in practical application, mostly through using high pressure sodium lamps (HPS) in the Northern Hemisphere (mainly Northern Europe). In the present study, we have focused on the quality of supplemental light for the production of rose-cut flowers. It has been shown that by the increase in the light intensity through the supplemental light, it is possible to accelerate the rate of growth and development of plants^6^. In accordance, with the current experiment, the highest amount of biomass in both cultivars of roses was related to plants exposed to supplemental light.

The combination of R and B light is an efficient light source for crop production in CEA^29^. In the previous studies, the importance of using R and B light spectra on the photosynthesis and growth of horticultural crops is highlighted^30^. R light promotes plant growth and root development^31^. Similar to the result of the present study, it has been reported that R light has a greater effect than B light on the growth of green and purple basil plants^32^. In the present study, among the supplemental light treatments tested, increasing the ratio of R to B light led to an increase in the vegetative growth of both rose cultivars, leading to improvement in the use of light. The SLA in both cultivars increased in Control and the thickness of their leaves decreased in a way to compensate for the shortage of light. Due to sufficient light intensity, plants restricted their leaf surface development under supplemental light treatments. Instead, they increase the thickness of their leaves and the density of photosynthesizing cells increases, and as a result, light losses are reduced^33^. Similar results have been reported by Hoshino^34^ in arabidopsis and Ghorbanzadeh^21^ in lettuce. Similar to our result, an increase in biomass as the result of supplemental light application has been reported in roses as well^35^. Increases in height and biomass^36^, leaf surface, and stem length^14^ by R light have been also reported. In the present experiment, the length of those shoots that were bent (for photoassimilate support) the surface of the leaf, as well as the biomass increased with having a higher ratio of R to lightweight. Studies showed that the combination of R and B light causes chloroplast development, biomass gain, and secondary metabolite production in tomatoes^37^. Similarly in basil plants, R light increased stem diameter, plant height, shoot dry weight, and number of leaves^32^.

### Supplemental light with a high ratio of R light accelerated flowering and improved yield of roses

Light is an effective agent for the opening of flower buds. In the absence/shortage of light, terminal and side buds opening would be inhibited/delayed in rose plants^38^. Increasing light intensity promotes flower induction and growth and decreases flower bud sterility in roses^6,38^. In accordance, with the present study, increasing the ratio of R to B light, led to great improvements in reproductive organ development in both rose cultivars. Flowering time was accelerated and flower quality was improved by the application of supplemental light in both studied cultivars. Previous experiments also showed that the application of supplemental light increased the blooming of roses^39^. The number of leaves and source organs are important factors for flowering. Therefore, R light improved the growth of the leaves, by providing more source organs for carbohydrate production, which is important for rose flowering. Furthermore, increasing the ratio of R to B light has accelerated flowering in the present study. It has been shown that R light plays a role in the development of flowering^38^.

**Figure 16:**
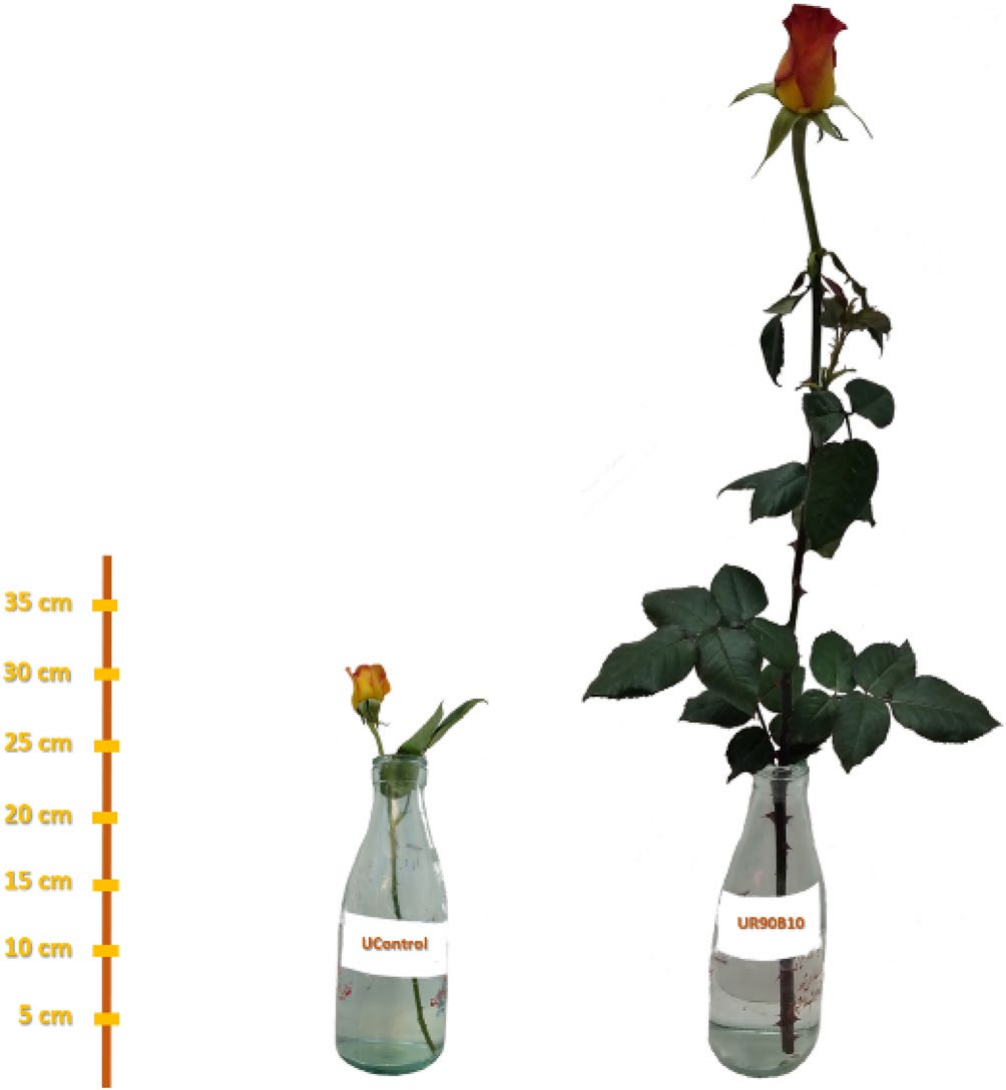
The difference in height of the rose branch of a rose cultivar (ʻUtopiaʼ) under the influence of R90B10 and control treatments.

Improving the effect of supplemental lighting, especially during periods when solar radiation is limited, on the production of more flower stems has been previously reported^38^. As a result, control treatment without supplemental light gained less biomass for flower production in both cultivars. Therefore, plant that did not reach an optimal amount of light, performs weaker in flowering (Fig. 16).

### Chlorophyll and carotenoid levels increased by a higher ratio of B spectrum, while carbohydrate and anthocyanin contents increased by a higher ratio of R spectrum of the supplemental light

In the current research, since the leaves of the bent shoots are the source of support for rose production, the biochemical properties were investigated in those leaves. Chlorophyll concentration increased in the control treatment without supplementary light. In low-light conditions (higher than minimum light intensity threshold that induce shade avoidance responses), plants increase their chlorophyll concentration to compensate for the lack of light^40^. Therefore, an increase in chlorophyll concentration is an adaptive strategy to tolerate light low-light conditions. An increase in carbohydrates was detected in the supplemental light treatment in the present study, especially with an increase in the ratio of R to Blight. Carbohydrates are one of the most important photosynthetic products that play a role in providing the cytoskeleton for growth and also for the osmotic regulation of plants^41^. Accumulation of soluble carbohydrates in rose leaves has been reported under R light; while B light reduced the carbohydrate concentration in the leaves^42^. Anthocyanin content is elevated by increasing the ratio of R to Blight. In a report on two cultivars of green and red-leaf cabbage, it was shown that in red-leaf cabbage, R light increased anthocyanin content, although there was no difference in their chlorophyll content, while in green-leaf cabbage, anthocyanin content regardless of the light quality was the same and the chlorophyll content was higher under B and blue-green light^43^. Increases in anthocyanins content^42,44^ and storage carbohydrates^44^ by R light have been reported in rose and savory plants. Furthermore, an increase in the amount of carotenoids and soluble carbohydrates by R light has been also reported in chrysanthemum cuttings^45^. The findings on the reproductive characteristics showed that by using supplemental light, marketability can be increased, which is more highlighted in plants exposed to R90B10 supplemental light treatments.

### Photosynthetic performance promoted by a high ratio of R light in the supplemental light

Our results showed that the best performance of the photosynthetic apparatus was detected under the combination of red and blue light treatments compared to the control, which is in according to the previoua reports^1^. In general, parameters obtained from chlorophyll fluorescence analysis (except for the PI_abs_) were increased in control plants. F_0_ increased in the plants receiving no supplemental light compared to those exposed to supplemental light treatments. The high F_0_ indicates the lack of proper functioning of the photosystems and the closedness of the reaction centers of photosystem II, which inhibits the transfer of electrons from Q_A_ to Q_B_ and reduces the function of trapping energy in photosystem II^46^. Regarding the V_j_, the low rate of electron transfer to the other side of quinone A is caused by the increase in fluorescence intensity in stage J of OJIP^47^ the decrease in J value is one of the main reasons for the reduction of PI_abs_^48^. V_j_ was the highest in the treatment without supplemental light. V_j_ shows information related to the number of closed reaction centers compared to the total number of reaction centers that can be closed.

PI_abs_ indicates the function of photosystem II and the pavement of electrons towards the photosystem I. Down-regulation of PI_abs_ can be due to the inhibition of electron transfer as the result of a decrease in the useful function of photosystem II^49^. The decrease in PI_abs_ is the result of high light energy absorption (ABS/RC), high rate of electron capture (TR_0_/RC), and high energy lost per reaction center (DI_0_/RC). In a report on the effect of light spectrum on high light stress in two cultivars of roses, a positive relationship was observed between soluble and storage carbohydrates and the PI_abs_ index. In a way the increase in photosynthesis performance is by high amount of carbohydrates^42^.

The high ABS/RC parameter can be the result of the inactivity of some reaction centers and a decrease in the reduction of quinone A^50^ Therefore, high ABS/RC in control plants is indicative of lack of proper function of some of their photosystem II. The performance index (PI) shows the energy exchange between the photons absorbed by photosystem II and the electronic regeneration of intersystem receptors and end receptors of photosystem I. It is also one of the very useful biophysical parameters in showing the difference between the response of photosystem II to stressful conditions and normal conditions^51^. PI_abs_ examines the three main steps namely absorption of light energy (ABS), trapping of light energy (TR), and conversion of excited energy to electron (ET) that drive photosynthetic activity by the photosystem II complex^49^. A high rate of TR_0_/RC indicates inhibition of QA^-^ to QA oxidation. As a result, the amount of electron transfer per reaction center decreases^52^. The increase in DI_0_/RC in the plants that received no supplemental light indicates that some photosystem II reaction centers have turned into silent reaction centers. These centers cannot regenerate quinone A, as a result, the quinone excitation energy is lost as heat^53^.

## Conclusions

The intensity, duration, and quality of light are the main factors affecting the growth and development of plants. Rose commercial production usually starts by maintaining enough shoots and leaves in a bending state to provide enogh carbohydrates needed to turn rose plants into the reproductive stage. This was quickly achieved when plants were exposed to supplemental lights. Among the treatments, plants exposed to R90B10 supplemental light showed the highest biomass and positive effects on flowering. The number of harvested flower shoots increased by application of supplemental light, which were more pronounced under R80B20 and R90B10 light treatment for both studied cultivars. The emergence of first buds was detected in R90B10 at the earliest time. R80B20 treatment decreased the length of the petals because the R80B20 treatment had a much shorter interval between the emergence of all flower buds than the other treatments. The height of the stem increased through the application of supplementary light compared to the treatment without supplemental light. Plants exposed to R90B10 treatment had the longest stems. Therefore, to improve the rose growth in the winter season, to increase the quality and quantity of flower branches, to accelerate flowering and marketability, the use of supplemental light with a high ratio of R light is an effective approach to improve rose production and quality.

### Permission was obtained to collect plant by mentioning the statement

Two grafted commercial rose cultivars (ʻSamuraiʼ and ʻUtopiaʼ) propagated through the stenting method using *Rosa canina* as the scion part free from the virus were obtained from research greenhouse of Sharekord University, Shahrekord, Iran. Rooted samples were cultured in pots with three volumetric units of cocopeat and one unit of perlite and placed in the research greenhouse of Horticulture Department, College of Agricultural Technology (Aburaihan), University of Tehran. The plant collection and use were in accordance with all the relevant guidelines. Regarding who did the official identification of the plant materials used in this study, it should be noted that all the cases were done under the supervision of the Corresponding author of this research.

### Supplementary Information

Supplementary Figure S1.

## Data Availability

The datasets used and/or analysed during the current study are available from the corresponding author on reasonable request. All data generated or analyzed during this study are included in this published article [and its supplementary information files].
