# Supplementary material for: Optimizing supplemental light spectrum improves growth and yield of cut roses
Source: Sci Rep. 2023 Dec 4;13:21381. doi: 10.1038/s41598-023-48266-3 (PMC10696034; doi:10.1038/s41598-023-48266-3)
Supplement: Supplementary file 1 — Supplementary Figure S1. [file 41598_2023_48266_MOESM1_ESM.docx]

**Supplementary Materials: Optimizing supplemental light spectrum improves growth and yield of cut Roses**

**Supplementary Materials**

Figure S1. Effect of different ratios of R and B light spectrum as supplemental light on PIABS (Performance index for the photochemical activity) (A), ABS/RC (The specific energy fluxes per RC for energy absorption) (B), TRo/RC (Trapped energy flux (leading to QA reduction) per RC) (C), ETo/RC (Electron transport flux (further than QA-) per RC) (D) and DIo/RC (Electron transport flux (further than QA-) per RC) (E) of two rose cultivars (ʻSamuraiʼ and ʻUtopiaʼ). Supplemental light recipes including 90% R: 10% B (R90B10), 80% R: 20% B (R80B20), 70% R: 30% B (R70B30), and treatment without supplemental light (Control) was used. Each column is representative of the mean value of six replicates plus SEM. Significance at the 0.05, 0.001, and 0.0001 probability levels are indicated by *, ***, and ****, respectively.


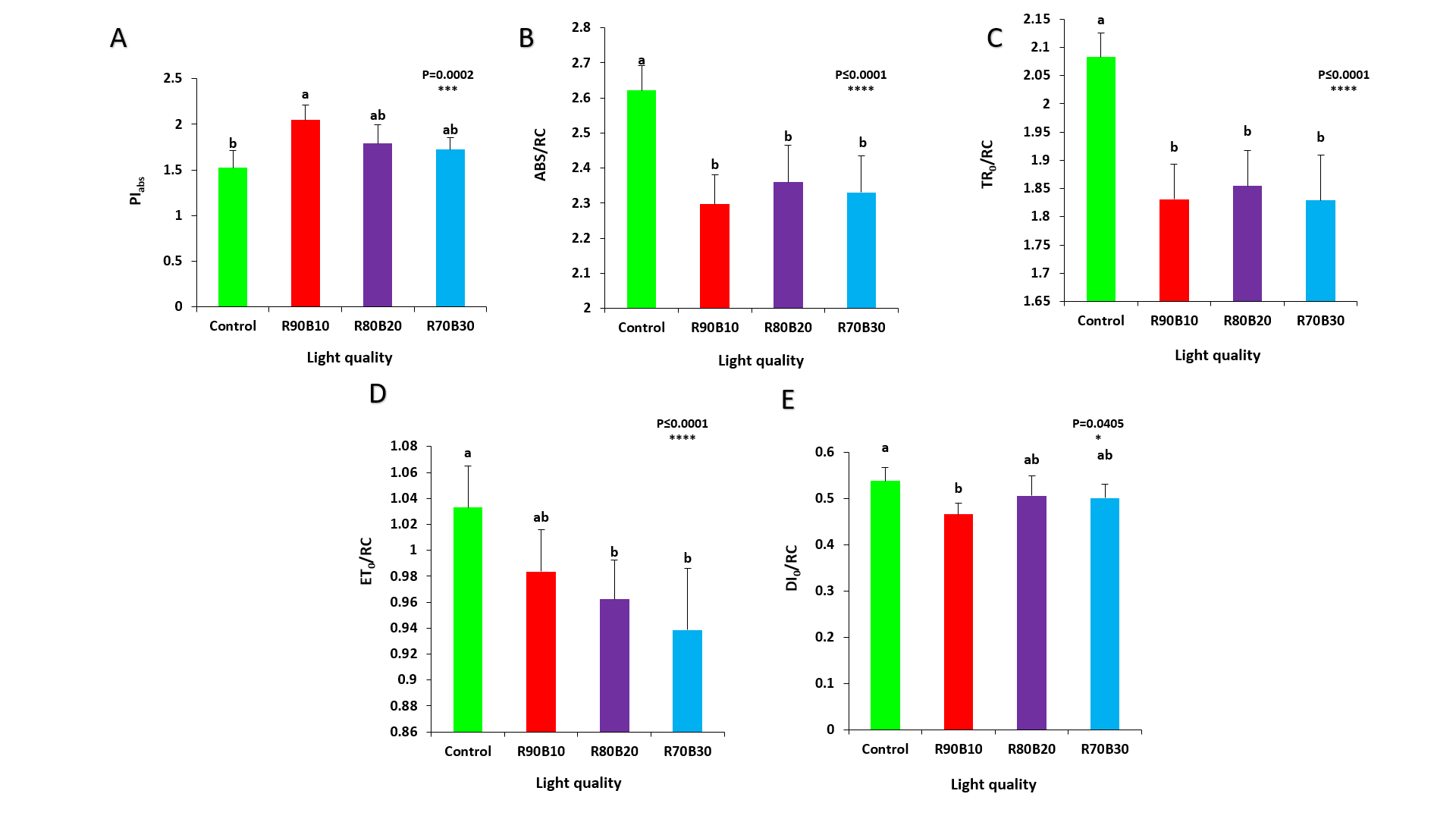


**Figure S1.** Effect of different ratios of R and B light spectrum as supplemental light on PI_ABS_ (Performance index for the photochemical activity) (A), ABS/RC (The specific energy fluxes per RC for energy absorption) (B), TR_0_/RC (Trapped energy flux (leading to Q_A_ reduction) per RC) (C), ET_0_/RC (Electron transport flux (further than Q_A_^-^) per RC) (D) and DI_0_/RC (Electron transport flux (further than Q_A_^-^) per RC) (E) of two rose cultivars (ʻSamuraiʼ and ʻUtopiaʼ). Supplemental light recipes including 90% R: 10% B (R90B10), 80% R: 20% B (R80B20), 70% R: 30% B (R70B30), and treatment without supplemental light (Control) was used. Each column is representative of the mean value of six replicates plus SEM. Significance at the 0.05, 0.001, and 0.0001 probability levels are indicated by *, ***, and ****, respectively.
